# PCF Based Sensor with High Sensitivity, High Birefringence and Low Confinement Losses for Liquid Analyte Sensing Applications

**DOI:** 10.3390/s151229891

**Published:** 2015-12-16

**Authors:** Huseyin Ademgil, Shyqyri Haxha

**Affiliations:** 1 Department of Computer Engineering, European University of Lefke, Mersin 10, Turkey; hademgil@eul.edu.tr; 2 Department of Computer Science and Technologies, University of Bedfordshire, University Square, Luton, Bedfordshire LU1 3JU, UK

**Keywords:** evanescent sensing, photonic crystal fiber based, fiber sensors, integrated optics

## Abstract

In this paper, we report a design of high sensitivity Photonic Crystal Fiber (PCF) sensor with high birefringence and low confinement losses for liquid analyte sensing applications. The proposed PCF structures are designed with supplementary elliptical air holes in the core region vertically-shaped V-PCF and horizontally-shaped H-PCF. The full vectorial Finite Element Method (FEM) simulations performed to examine the sensitivity, the confinement losses, the effective refractive index and the modal birefringence features of the proposed elliptical air hole PCF structures. We show that the proposed PCF structures exhibit high relative sensitivity, high birefringence and low confinement losses simultaneously for various analytes.

## 1. Introduction

Fiber optic technology was primarily developed for telecommunication applications. However, due to the advances in fabrication technology optical fibers have also contributed to the expansion of guided wave technology for sensing applications. The development of photonic crystal fibers (PCFs) and their large number of potential applications have demonstrated the potential virtues of optical fibers in chemical and biological sensing [1,2,3,4,5,6]. The outstanding characteristics of these microstructured fibers such as small size, potential for remote and continuous sensing, freedom from electrical interference, and relative compatibility with fiber optic telecommunications technology, make them stand out for sensing applications [1,2,3,4,5,6]. Conventional step index fibers have strict design rules to satisfy, such as limited core size for single-mode operation, modal cut off wavelength and limitation on material selection as the core and cladding materials must have matching thermal properties. Moreover, restrictions on the geometry hinder the flexibility in realizing fiber properties such as dispersion [7], nonlinearity [8] and birefringence [9] for better performance and more specialized applications.

The developing manufacturing technology of PCFs allows us to achieve exceptional propagation properties by choosing the appropriate design parameters. Light propagation characteristics and inherited geometric flexibility of the PCFs allows us to achieve unconventional propagation properties by adjusting the air holes in the core and cladding region. Various research studies of these mirostructured fibers have demonstrated that outstanding dispersion properties [7,10], endlessly single-mode guidance [11], light guidance in lower index material [12], high birefringence [9,10] and enhanced nonlinear effects [8] can be achieved for wide wavelength ranges. These unique light properties of PCFs have contributed to further development of these structures in various applications in the fields of optical communications [7], nonlinear optics [8], high power technology [13] and sensing [1,2,3,4,5].

The availability of low loss optical fibers with the associated benefits of small size, low cost, design flexibility, robustness and wide bandwidth has led to the development of fiber-based sensors. Conventional step index-based optical sensors are commercially available [14], while PCF-based sensor devices are relatively novel and are still in the development stage, but with a great potential for contributing to wider application areas.

Hollow core Photonic Band Gap (PBG) PCFs can be an ideal candidate for sensing applications [4,5] where, the direct interaction of the light and the sample within the hollow fiber core is higher than index-guided PCFs. These types of fibers are more convenient for gas sensing applications. Conversely, for liquid sensing applications, PCFs with index guiding mechanism are desirable. The sensing mechanism of index-guiding PCF sensors is based on the evanescent interaction between the guided optical field and the sample, similar to that in the conventional sensors.

The evanescent field associated with the light propagating in the confinement region of the device extends into the region where the analyte to be sensed is located. The presence of air-holes in the cladding microstructure allows the accommodation of biological and chemical samples in gaseous or liquid forms in the immediate locality of the fiber core [6,15,16].

In recent years, the idea of filling the PCF core or cladding holes with various analytes (gases and/or liquids) has attracted much research attention. The advancements offered by the PCFs are becoming readily available for the measurement of many physical and chemical parameters, such as temperature, pressure, liquid levels and composition. These features are making PCF-based sensors ideal candidates for chemical and biological sensing of low index materials [4,5,17,18]. Since PCF-based sensors permit substantial interaction between the light guided along the fiber and any chemical species situated in the holes, they can be used for remote sensing and bacteria detection [6,18,19,20]. Water and alcohols are considered as the major analytes for these types of applications because they account for the immense majority of biological or chemical solutions.

As mentioned earlier, the typical process for sensing the low refractive index analytes depends on the interaction of the evanescent field of the guiding mode with the analyte (gas or liquid) under investigation (detection) [6,16]. It is worth stating that the evanescent PCF sensor is an intrinsic sensor, since the core of the PCF interacts directly with the material being analyzed. A PCF-based sensor typically consists of a transparent core surrounded by cladding of lower refractive index (air holes). The light is retained inside the core based on the total internal reflection, however the internally reflected light actually penetrates, for a small distance, into the cladding region. In an evanescent PCF sensor, the key design parameter is the air hole size and the hole to hole distance that forms the cladding area. In the past, a number of research studies has been carried out for investigating the propagation features of PCFs and the potential applications against various analytes (gases and/or liquids) [4,6,16]. To the extent of our knowledge, evanescent PCF sensing, in which both cladding and core are microstructured has been introduced by Monro *et al*. [15]. Subsequently, Cordeiro *et al*. [6] have theoretically and experimentally studied numerous PCF structures with various analytes filled in the core holes. Further, improvement of the relative sensitivity with lower losses has been achieved with octagonal PCF structure in [21]. Yu *et al.* [22] has demonstrated ultrasensitive PCF-based temperature sensors, where core holes are filled with ethanol. The authors in [22] have shown that the proposed sensor is simple and convenient to use and it can be produced at a low cost. Additionally, Liu *et al.* [17] have proposed a PCF structure that can be used for water level sensing and environmental temperature sensing.

In some specific fiber optic sensors, preserving the state of polarization in fiber is both important and challenging. Conventional step index fibers with perfectly circular cores cannot maintain the polarization state of the electromagnetic field due to several factors, as stated in [23]. The polarization state of the optical field can be retained by introducing birefringence. Highly birefringent fibers can be used as active elements of fiber optic sensor configurations utilizing the interference of polarization modes [24]. The most common techniques to achieve birefringence in PCFs is to alter the air hole size [9] or by distorting the shape of the air hole (elliptical air holes) [9,10] around the PCF core. To our knowledge, structures introduced by Kim *et al*. [10] have demonstrated that relatively large birefringence in the order of 10^−3^–10^−2^ can be achieved with PCFs structures.

It is important to emphasize that, unlike traditional polarization-maintaining fibers [25], which comprise at least of two types of glass material, each one with a different thermal coefficient, the birefringence obtainable with PCFs is highly insensitive to temperature, which is a significant feature for various sensing applications, as it is well known that the temperature cross-sensitivity affects the measurement accuracy of the optical sensors. Therefore, in order to overcome this limitation, birefringent PCFs have emerged as active elements of the optical sensors. As described in [24], these types of fibers are suitable for hydrostatic pressure sensing. Tuneable high birefringence PCFs can be obtained by symmetrically filling the sensitive materials into air holes. As a new type of functional material, magnetic fluids seem to be particularly interesting substances to infiltrate PCFs, since their refractive index is sensitive to external magnetic field [25].

Furthermore, highly birefringent with a high sensitivity PCFs have been investigated for various sensing applications [26,27]. In [26], a strain and temperature sensitive birefringent PCF was designed by using Ge-doped silica rods on the *x*-axis of the PCF structure. On the other hand, birefringence in a polarization-maintaining PCF as a function of the temperature is demonstrated experimentally in [27].

In this research work, the evanescent hexagonal PCF structure with elliptical cladding and core holes is investigated. In this design, in order to achieve high birefringence, high sensitivity and low confinement losses simultaneously, elliptical air holes have been constructed in the cladding and in the core region. Previously, Kim *et al.* [10] have theoretically demonstrated that simultaneously high birefringence with dispersion control can be realized with elliptical air holed PCF structure. To the extent of our knowledge, there are no published research papers that have studied and optimized the sensitivity performance of PCFs with elliptical air holes. In this paper, the well-known technique implemented by [6] is applied to an elliptical air holed PCF structure. In order to realize the effects of elliptic air holes more efficiently, vertical and horizontal air hole configurations are introduced and analyzed. The relative sensitivity and the birefringence of the proposed PCF structures against specific liquid analytes are investigated and compared thoroughly. Furthermore, effects of the operating wavelength and the ellipticity of the cladding holes are studied for the proposed PCF structures.

## 2. Design, Numerical Results and Discussion

In this study, full vectorial Finite Element Method (FEM) with the perfectly match layer (PML) boundary condition are applied, which is one of the most powerful numerical approaches available to engineers for designing and developing photonic components and devices [10,15,16,18,19]. The PML as boundary conditions is a useful technique to evaluate propagation characteristics of leaky modes in PCFs and by applying these layers, all optical propagation properties can be evaluated in a single run [7,28]. The modal analyses have been made on the cross-section in the *x*-*y* plane of the PCF as the wave is propagating in the z-direction.

Maxwell’s equations with the anisotropic PML boundary conditions [7,21] can be expressed as:

(1) $$\nabla \times ({\left[\text{s}\right]}^{-1}\nabla \times E-k_{0}^{2}n^{2}\left[s\right]E=0$$

where *E* is the electric field vector, *k*_0_ (= 2*π*/*λ*) is the wave-number in the vacuum, *n* is the refractive index of the domain, [s] is the PML matrix, [s]^−1^ is an inverse matrix of [s] and *λ* is the operating wavelength. To understand the advantages of the proposed PCF for sensing applicatins and its performance, it is highly important to evaluate the relative sensitivity coefficient *r* and sensitivity *f*, which are expressed by the modified *Beer Lambert Law,* expressed as [6]:

(2) $$r=\frac{n_{r}}{n_{e}}\;\times f$$

where, *n_r_* is the refractive index of the absorbing material and *n_e_* is the real part of effective refractive index *f* is the the percentage of energy presents in the holes. According to Poynting’s theorem, the effective refractive index *f* can be expressed as [6]:

(3) $$f=\frac{\left(sample\right)\int^{\text{​}}Re\left(E_{x}H_{y}^{*}-\;E_{y}H_{x}^{*}\right)dxdy}{\left(total\right)\int^{\text{​}}Re\left(E_{x}H_{y}^{*}-\;E_{y}H_{x}^{*}\right)dxdy}\times 100$$

**Figure 1:**
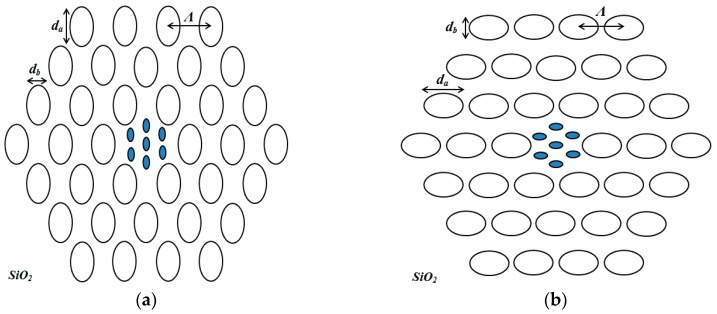
(**a**) Schematic depiction of the Vertical air holed PCF (V-PCF) and (**b**) Horizontal air holed PCF (H-PCF).

Schematics of the proposed PCF sensor structure configuration are ilustrated in Figure 1a,b. In these figures we show the proposed PCF with elliptical air holes in the outer region and supplementary elliptical air holes in the core region. The elliptical air holes in the PCF structures are designed vertically, V-PCF (Figure 1a) and horizontally, H-PCF (Figure 1b). The size of the cladding elliptic air holes are denoted by the ellipticity constant, *η.* The ellipticity constant is defined as *η* = *d_b_/d_a_* , where *d_a_* and *d_b_* are the lengths of the major and minor axes, respectively. The supplementary air holes are denoted by *d_c_* and *d_s_* and set to 0.2 µm and 0.4 µm, respectively. In our simulations, the hole to hole spacing in the core and in the cladding region is set to *Λ_core_ =* 1 µm and *Λ_cladding_* = 2.4 µm, respectively. Our proposed PCF structures contains 36 air holes in the cladding region and the index of refraction of the cladding holes are set to 1. Silica with 1.45 index of refraction is used as a background material. This results in higher air filling ratio and lower refractive index around the core, thus providing strong confinement ability.

As it can be seen from Figure 1, the PCF structures accomodate the liquid filled in the supplementary core holes. The selected analytes used for sensing in the peoporsed V-PCF and H-PCF structures are: water, ethanol and benzyne with the refractive indices 1.333, 1.354 and 1.366, respectively [29]. Due to the small numerical aparture and small index difference between PCF core and cladding, the proposed PCF structures support the fundamental mode and some other higher order modes. These weakly guided modes are called *LP* modes, which are a linear combination of the degenerate exact modes. In this case the fundamental *LP*_01_ mode matches to *HE*_11_. In this work, only the fundamental *LP* modes are considered.

**Figure 2:**
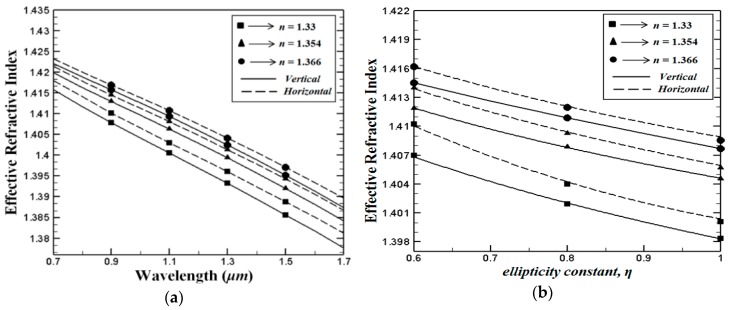
(**a**) Wavelength depending effective refractive index of the fundamental $HE_{11}^{x}$ mode, where *η* = 0.7, and (**b**) the effective refractive index of the fundamental $HE_{11}^{x}$ mode as a function of ellipticity constant *η*, where the wavelength is fixed to λ = 1.

Initially, the effective refractive index behaviour of the proposed PCF structures was studied. The effective refractive index of the fundamental *LP_01_* ($HE_{11}^{x}$) mode depending on wavelength for proposed PCF structures where, *η* = 0.7 (*d_a_ = 1* μm and *d_b_* = 0.7 μm*)* is presented in Figure 2a. The numerical calculations of the V-PCF and the H-PCF are indicated with solid and dashed curves, respectively. It can be evidently seen from the figure that, compared with the V-PCF structure, the H-PCF one displays higher effective index values over a wide variety of wavelengths. Our simulations show that the effective refractive index values are growing when the supplementary core holes are occupied with higher index analytes. For both PCF structures, an increase in the operating wavelength results in a linear decrease in the effective refractive index values. Furthermore, calculations in Figure 2b show that the effective refractive index values of both proposed structures are reduced with increasing ellipticity *η*, at λ = 1 μm. It is worth noting that the cladding air holes are perfectly circular when *η* = 1. The above results show that the effective refractive index can be controlled by either tuning the ellipticity of the cladding holes or by precisely selecting the analytes. This allows the light intensity distribution near the core and over the cladding region.

**Figure 3:**
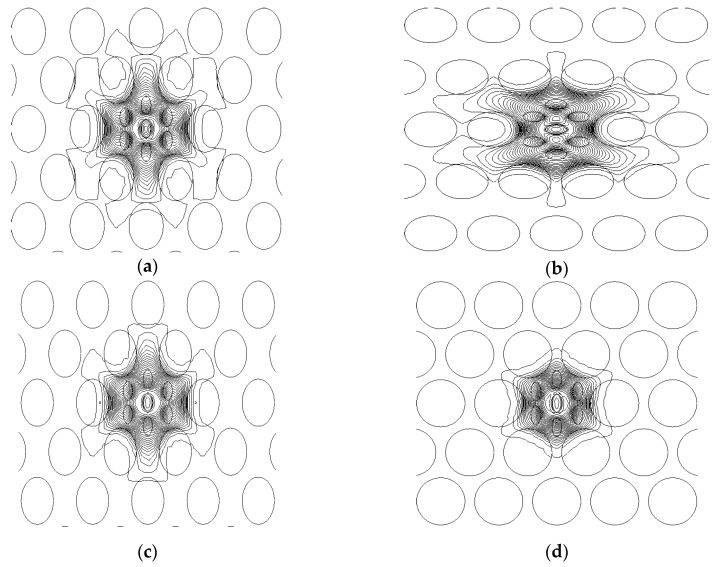
The electric field distribution of the *x*-polirized *LP_01_* mode of (**a**) V-PCF and (**b**) H-PCF, where *η* = 0.7 and the core holes are filled with refractive index of 1.33. The field profile of V-PCF where, supplementary holes are filled with (**c**) *n* = 1.366, *η* = 0.7, and (**d**) *n* = 1.366, *η* = 1 (for all figures the wavelenght is fixed at λ = 1 μm)*.*

Figure 3 shows the the electric field distribution of the x-polirized fundamental $HE_{11}^{x}$ mode of the proposed PCF (V-PCF (a) and H-PCF (b)) structures when the core holes are filled with analytes with refractive index 1.33 and 1.366. It can be clearly seen from Figure 3a,b that the electric fields are distributed in opposite axises where V-PCF and H-PCF structures are used, resulting in different field confinments. Furthermore, it can be seen when a higher refractive index analyte is used, the electric fields shown in Figure 3c,d are more confined than the electric fields shown for the PCF structures shown in Figure 3a,b when lower refractive index analyte is used. It should be mentioned that when a higher index analyte is used in the supplementary core holes, in this case this will lead to higher power interactions between the guided light and the analyte under investigation. Also, it can be seen that, due to planar waveguide channels of H-PCF structure the light leakage is slightly higher than V-PCF. Furthermore, it can be seen from the Figure 3d that the electric field distribution is almost similar on both axis where perfectly circular air holes are used in the cladding region. It is worth stating that this phenomena is evident for lower birefringence structures.

**Figure 4:**
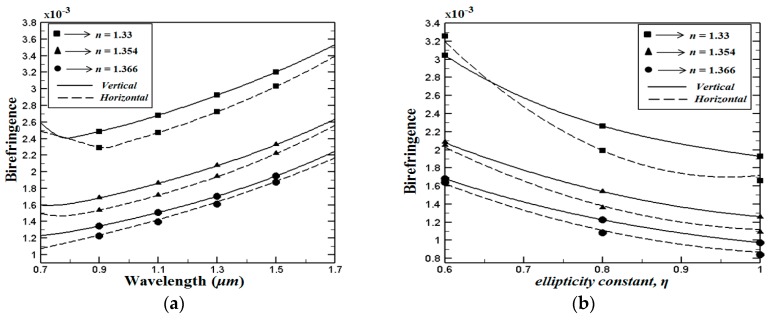
(**a**) Variation of the birefringence as the function of the wavelength, (**b**) variation of the birefringence as a function of the ellipticity constant *η*, where the operating wavelength is fixed to 1 μm.

Next, Figure 4a shows the wavelength dependent modal birefringence, where *η* = 0.7 (*d_a_ =* 1 μm and *d_b_* = 0.7 μm*)*. It can be seen from this figure that birefringence values on the order of 10^−3^ are obtained for both proposed PCF structures. Numerical results have indicated that the birefringence of the proposed V-PCF structures is higher than H-PCF structure. In addition, the birefringence values are increasing with the operating wavelength. Furthermore, Figure 4b shows the variation of the birefringence as a function of the ellipticity constant *η*, where operating wavelength is set to 1 μm. As expected, the birefringence of the proposed PCF structures is decreasing when the elipticity constant is increasing. The perfectly circular cladding air holes of the proposed structures have a significant high birefringence. This phonemona is directly related to the fact that the elliptical air holes in the core region are filled with the liquid.

**Figure 5:**
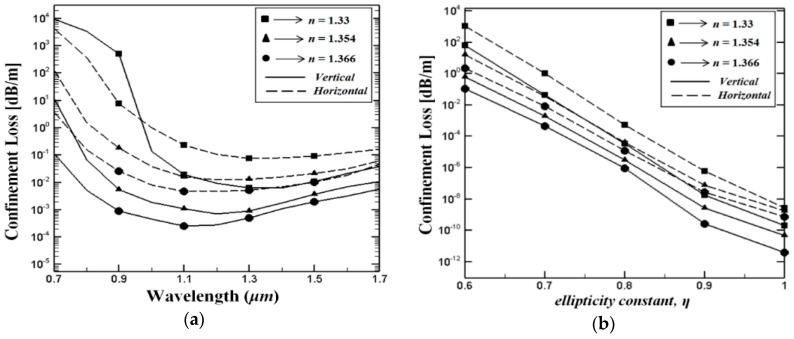
(**a**) Variation of the confinment losses as a function of the wavelength where *η* = 0.7 and (**b**) variation of the confinment losses as a function of the ellipticity constant *η*, where operating wavelength is fixed to 1 μm.

It is already known that the mode confinement of guided light is a key parameter in the propagation properties of PCF-based devices. In evanescent PCF models, since the interraction between the guided light and the analyte is critical, the evaluation of the confinement losses needs to be studied intensely. The fundamental design parameters that are decisive on the PCF confinement losses are: the number of air hole rings in the cladding, air hole sizes and the distance between these holes. Furthermore the core size and the operating wavelength are also essential design parameter. Figure 5a shows wavelength dependent confinement loss where *η* = 0.7 (*d_a_ = 1* μm and *d_b_* = 0.7 μm). The numerical results indicate that the confinement losses of the proposed PCF structures vary for each analyte. This phenomenon can be confidentally linked to the refractive index of the liquids filled in the supplementary core holes. Further, it can be noticed that the field confinement improves with higher index liquids for both proposed structures over a wide wavelength range. In addition, at shorter wavelengths the confinement losses are reducing gradually and become steady at longer wavelengths. On the other hand, Figure 5b shows the effect of the ellipticity *η* on the confinement losses, where the wavelength is fixed to *λ* = 1 µm. This figure indicates that the confinement losses of both PCF structures are linearly reducing with increasing of the ellipticity constant *η*. In addition, lower confinement losses are achieved with higher index liquids. 

By investigating Figure 4 and Figure 5 together, it can be seen that V-PCF structure exhibits better performance in terms of birefringence and confinement losses for all types of the analytes used in this study. In other words the V-PCF structure displays lower confinement losses and higher birefringence than the H-PCF structure. These findings can be linked to the planar waveguide channels created by the H-PCF in the cladding region (see Figure 1b) which instigates the light to leak out from the core region. 

**Figure 6:**
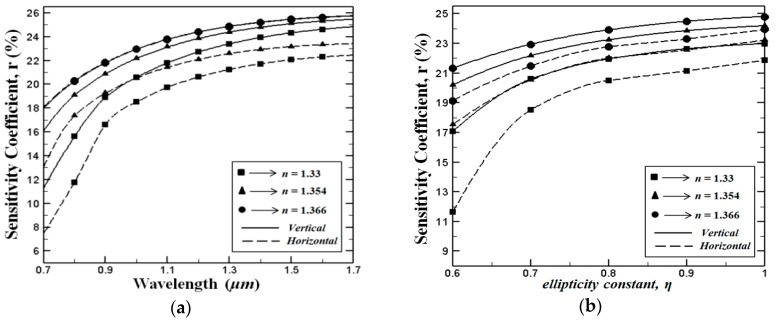
(**a**) Variation of the sensitivity coefficient *r* as a function of the wavelength when *η* = 0.7 and (**b**) variation of the sensitivity coefficient *r* as a function of the ellipticity constant *η*, when the wavelength is fixed to 1 μm.

Together with the modal birefringence and confinement losses, the relative sensitivity coefficient *r*, which indicates the light analyte interraction percentage, is one of the most vital parameters for PCF-based sensors. As presented earlier in Figure 5 when compared to the H-PCF structure the fundamental mode of the V-PCF structure is more confined in the PCF core region. Therefore, the light analyte interaction in the V-PCF structure is expected to be higher than in the H-PCF structure. In Figure 6a we show the wavelength dependent relative sensitivity *r* for various analytes, where *η* = 0.7 (*d_a_ =* 1 μm and *d_b_* = 0.7 μm*).* As expected, our simulation results indicate that the relative sensitivity values of the V-PCF structure are fairly higher than those for the H-PCF structure. As can be seen from the figure, at longer wavelengths the relative sensitivity coefficient is increasing for all analytes in both cases (V-PCF and H-PCF). This phenomenon can be related to a higher electromagnetic power interaction between light and the analyte filled in the supplementary core holes. Furthermore, we illustrate the variation of the sensitivity coefficient *r* as a function of the wavelenght (Figure 6a) and variation of the sensitivity coefficient as a function of the ellipticity constant *η* when the wavelength is fixed to *λ* = 1 µm (Figure 6b). As can be seen, our simulation demonstrate that for all analytes and design parameters higher sensitivity levels are obtained for the proposed V-PCF structure. It is worth noting that the highest relative sensitivity coefficient for both proposed PCF are achieved where *η* = 1*.*

Simultaneously achieving high sensitivity, low confinement loss and high birefringence over a wide wavelength range is essential for many optical sensing applications. In this work, we have shown that all these propagation features can be achieved in our proposed PCF (V-PCF and H-PCF) structures. The trade off between high birefringence and high sensitivity coefficient can be realized from the numerical calculations. Our numerical results indicate that both features are acceptable at level *η* = 0.7–0.8.

Finally, from the view of fabrication feasibility and application, it is essential to analyze and envisage the manufacturing issues of the proposed PCF structures. It is worth noting that the suggested PCF structures might not be easy to manufacture. However, with the innovative advances in the production of PCFs, the authors are convinced that the fabrication of the recommended PCF structures is possible. The fiber holes must be filled with analyte/liquid in a way that does not destruct the fiber's functionality and the laser light must be as close to single mode as possible to achieve the required sensitivity.

There are several techniques that can be used for selectively filling the PCF holes with analytes. It is worth revealing that in our proposed PCF structure, in terms of analyte filling, the complexity is increased slightly primarily in the core area. However, Huang *et al*. [30] have established a unique method which allows for selectively filling the air holes in air-core PCFs and forming various functional PCFs for different applications. More specifically, authors have demonstrated that it is possible to fill any cladding hole or just the supplementary core holes. The filling process comprises of pressurizing UV-curable polymer inside the PCF. More recently, Luo *et al*. [31] and Gerosa *et al*. [32] have experimentally shown that fabrication of the PCF structures with liquid filled cladding/core holes can be efficiently accomplished by using similar methods. In this regard our proposed PCF structure can be fabricated with the current available nanotechnology, and it exhibits significant simultaneous advantages in terms of key sensor parameters such as high birefringence, high sensitivity and low confinement losses.

## 3. Conclusions

A technique applied by [6] and recently studied by [21] has been effectively applied to index guided elliptical holed PCF structures. The effects of the ellipticity constant and the operating wavelength have been studied for selected liquids. Our theoretical study has shown that it is possible to simultaneously achieve high birefringence, and low confinement losses with high sensitivity for various analytes. Also, simulation results have demonstrated that the overall performance of V-PCF structure is superior than H-PCF structure for all tested liquids. In this study, the key PCF design parameters have been fully optimized in order to increase the manufacturing efficiency. In this regard, authors believed that, with the current advances in nanofabrication techniques, manufacturing of the proposed PCF structures is possible [33]. As a result, the proposed PCF structures offer great potential for liquid analyte sensing, making the proposed PCF structures a good candidate for future applications in chemical and biological sensing.

## References

[1] Zheng S., Shan B., Ghandehari M., Ou J. (2015). Sensitivity characterization of cladding modes in long-period gratings photonic crystal fiber for structural health monitoring. Measurement.

[2] Zheng S., Zhu Y., Krishnaswamy S. (2013). Fiber humidity sensors with high sensitivity and selectivity based on interior nanofilm-coated photonic crystal fiber long-period gratings. Sens. Actuators B Chem..

[3] Zhu Y., Du H., Bise R. (2006). Design of solid-core microstructured optical fiber with steering-wheel air cladding for optimal evanescent-field sensing. Opt. Express.

[4] Cubillas A.M., Unterkofler S., Euser T.G., Etzold B.J.M., Jones A.C., Sadler P.J., Wasserscheid P., Russell P.St.J. (2013). Photonic crystal fibres for chemical sensing and photochemistry. Chem. Soc. Rev..

[5] Pinto A.M.R., Baptista J.M., Santos J.L., Lopez M.A., Frazão O. (2012). Micro-displacement sensor based on a hollow-core photonic crystal fiber. Sensors.

[6] Cordeiro C.M.B., Franco M.A.R., Chesini G., Barretto E.C.S., Lwin R., Cruz C.H.B., Large M.C.J. (2006). Microstructured-core optical fibre for evanescent sensing applications. Opt. Express.

[7] Saitoh K., Koshiba M., Hasegawa T., Sasaoka E. (2003). Chromatic dispersion control in photonic crystal fibres: Application to ultra-flattened dispersion. Opt. Express.

[8] Hansen K. (2003). Dispersion flattened hybrid-core nonlinear photonic crystal fiber. Opt. Express.

[9] Steel M.J., Osgood R.M. (2001). Elliptical hole photonic crystal fibers. Opt. Lett..

[10] Kim S.E., Kim B.H., Lee C.G., Lee S., Oh K., Kee C.S. (2012). Elliptical defected core photonic crystal fiber with high birefringence and negative flattened dispersion. Opt. Express.

[11] Birks T.A., Knight J.C., Russell P.S.J. (1997). Endlessly single-mode photonic crystal fiber. Opt. Lett..

[12] Argyros A., Eijkelenborg M.A.V., Large M.C.J., Bassett I.M. (2006). Hollow-core microstructured polymer optical fiber. Opt. Lett..

[13] Limpert J., Limpert T., Schreiber S., Nolte H., Zellmer T., Tunnermann R., Iliew F., Lederer J., Broeng G., Vienne G. (2003). High-power air-clad large-mode-area photonic crystal fiber laser. Opt. Express.

[14] Lee H.W., Schmidt M.A., Uebel P., Tyagi H., Joly N.Y., Scharrer M., Russell P.St.J. (2011). Optofluidic refractive-index sensor in step-index fiber with parallel hollow micro-channel. Opt. Express.

[15] Monro T.M., Belardi W., Furusawa K.J., Baggett C.N., Broderick G.R., Richardson D.J. (2001). Sensing with microstructured optical fibres. Meas. Sci. Technol..

[16] Hoo Y.L., Jin W., Ho H.L., Wang D.N., Windeler R.S. (2002). Evanescent-wave gas sensing using microstructure fiber. Optical Eng..

[17] Liu S., Wang Y., Hou M., Guo J., Li Z., Lu P. (2013). Anti-resonant reflecting guidance in alcohol-filled hollow core photonic crystal fiber for sensing applications. Opt. Express.

[18] Kuhlmey B.T., Eggleton B.J., Wu D.K.C. (2008). Fluid-filled solid-core photonic bandgap fibers. J. Lightwave Technol..

[19] Vieweg M., Gissibl T., Pricking S., Kuhlmey B.T., Wu D.C., Eggleton B.J., Giessen H. (2010). Ultrafast nonlinear optofluidics in selectively liquid-filled photonic crystal fibers. Opt. Express.

[20] Konorov S.O., Zheltikov A., Scalora M. (2005). Photonic-crystal fiber as a multifunctional optical sensor and sample collector. Opt. Express.

[21] Ademgil A. (2014). Highly sensitive octagonal photonic crystal fiber based sensor. Optik Int. J. Light Electron Opt..

[22] Yu Y., Li X., Hong X., Deng Y., Song K., Geng Y., Wei H., Tong W. (2010). Some features of the photonic crystal fiber temperature sensor with liquid ethanol filling. Opt. Express.

[23] Sharma M., Borogohain N., Konar S. (2013). Index guiding photonic crystal fibers with large birefringence and walk-off. J. Lightwave Technol..

[24] Hlubina P., Martynkien T., Olszewski J., Mergo P., Makara M., Poturaj K., Urbanczyk W. (2013). Spectral-domain measurements of birefringence and sensing characteristics of a side-hole microstructured fiber. Sensors.

[25] Zhang Y., Li D. (2012). Analysis of Birefringent Characteristics of Photonic Crystal Fibers Filled Magnetic Fluid. Advances in Brain Inspired Cognitive Systems, Lecture Notes in Computer Science.

[26] Pang M., Xiao L.M., Jin W., Cerqueira A.S. (2012). Birefringence of hybrid PCF and its sensitivity to strain and temperature. J. Lightwave Technol..

[27] Ma P., Song N., Jin J., Song J., Xu X. (2012). Birefringence sensitivity to temperature of polarization maintaining photonic crystal fibers. Opt. Laser Technol..

[28] Selleri S., Vincetti L., Cucinotta A., Zoboli M. (2001). Complex FEM modal solver of optical waveguides with PML boundary conditions. Opt. Quantum Electron..

[29] Kamikawachi R.C., Abe I., Paterno A.S., Kalinowski H.J., Muller M., Pinto J. l., Fabris L. (2008). Determination of thermo-optic coefficient in liquids with fiber Bragg grating refractometer. Opt. Commun..

[30] Huang Y., Xu Y., Yariv A. (2004). Fabrication of functional microstructured optical fibers through a selective-filling technique. Appl. Phys. Lett..

[31] Luo M., Liu Y., Wang Z., Han T., Wu Z., Guo J., Huang W. (2013). Twin-resonance-coupling and high sensitivity sensing characteristics of a selectively fluid-filled microstructured optical fiber. Opt. Express.

[32] Gerosa R.M., Spadoti D.H., de Matos C.J.S., de Menezes S.L., Franco M.A.R. (2011). Efficient and short-range light coupling to index-matched liquid-filled hole in a solid-core photonic crystal fiber. Opt. Express.

[33] Issa N.A., Eijkelenborg M.A.V., Fellew M. (2004). Fabrication and study of microstructured optical fibers with elliptical holes. Opt. Lett..

